# Radiomic Assessment of Epicardial Adipose Tissue for the Prediction of Non-Calcified Coronary Atherosclerotic Plaques

**DOI:** 10.3390/jcdd13030113

**Published:** 2026-03-02

**Authors:** Carlo Di Donna, Armando Ugo Cavallo, Eliseo Picchi, Mario Laudazi, Massimo Federici, Marcello Chiocchi, Francesco Garaci

**Affiliations:** 1 Diagnostic Imaging Unit, Department of Biomedicine and Prevention, University of Rome Tor Vergata, 00133 Rome, Italy; didonnacarlo@gmail.com (C.D.D.); francesco.garaci@uniroma2.it (F.G.); 2 Radiology Unit, Sant’ Andrea University Hospital, 00189 Rome, Italy; 3 Department of Radiology, Istituto Dermopatico dell’Immacolata IDI-IRCCS, 00167 Rome, Italy; 4 Department of Systems Medicine, University of Rome Tor Vergata, 00133 Rome, Italy

**Keywords:** epicardial adipose tissue, radiomics, coronary artery disease, CAD-RADS, cardiac-CT

## Abstract

Epicardial adipose tissue (EAT) has previously been associated with coronary artery calcium scores, an increased burden of coronary artery disease (CAD), and features of plaque instability. These associations are likely mediated by endocrine and paracrine signaling from bioactive molecules secreted by EAT, which may contribute to coronary atherosclerosis. EAT can be non-invasively quantified on images obtained during coronary computed tomography angiography (CCTA). This study aimed to evaluate the potential association between EAT and non-calcified coronary plaques with severe stenosis using radiomic methodology. Materials and Methods: A total of 128 consecutive patients undergoing CCTA—both with and without contrast—for known or suspected CAD were retrospectively analyzed. EAT features were extracted from contrast scans. Coronary artery plaque features were evaluated using Coronary Artery Disease-Reporting and Data System (CAD-RADS). Results: EAT features showed a statistically significant positive correlation with non-calcified coronary plaques with severe grades of stenosis (CAD-RADS > 4). The Ensemble Machine Learning (EML) model combined with coronary plaque data showed a sensitivity of 1.00 and a specificity of 0.93, with a negative predictive value of 1.00 and a positive predictive value of 0.85, and an accuracy of 0.95 (95% CI: 0.9221–1) in internal validation. Conclusions: EAT may represent a novel imaging biomarker associated with the presence of actionable coronary plaques. Radiomic texture analysis of EAT could enhance the non-invasive prediction of coronary stenoses. These preliminary findings support the clinical utility of EAT evaluation via CCTA in patients with low to intermediate cardiovascular risk.

## 1. Introduction

### 1.1. Coronary Artery Disease

Coronary artery disease (CAD) is a chronic, often progressive condition caused by the buildup of atherosclerotic plaques in the coronary arteries, influenced by risk factors such as dyslipidemia, diabetes, hypertension, smoking, family history, and lifestyle [1]. While CAD can remain stable, it may suddenly become unstable due to acute atherothrombotic events [2]. Coronary computed tomography angiography (CCTA) plays a key role in diagnosing CAD in patients with low to intermediate risk, offering high sensitivity and specificity for detecting coronary stenosis and plaque vulnerability [3,4]. CCTA directly visualizes both stenosis and atherosclerotic plaque, making it more reliable than functional tests in ruling out CAD and reducing unnecessary invasive coronary angiography [5,6]. Advances such as CT-derived fractional flow reserve and CT perfusion enhance its diagnostic accuracy [7,8,9,10,11,12]. CCTA can assess plaque morphology, predict future cardiovascular events, and support preventive therapy decisions. With decreasing contrast and radiation burdens, CCTA is increasingly cost-effective and is now recommended as the first-line test for stable chest pain evaluation by guidelines like NICE [12].

### 1.2. Epicardial Adipose Tissue (EAT)

Epicardial adipose tissue (EAT) and its density (EAD) can be readily measured on CCTA scans [13,14]. Various studies have shown a correlation between EAT volume and EAD, as measured by CCTA, and factors such as coronary artery calcium score, coronary artery disease burden, and plaque vulnerability [15,16,17,18].

Epicardial adipose tissue primarily serves a protective role by providing mechanical support, supplying energy to the myocardium, and secreting anti-inflammatory adipokines. However, due to its potential for excessive lipid accumulation and expansion, EAT is currently considered a risk-enhancing factor for metabolic syndrome, similar to abdominal visceral adipose tissue [18].

Visceral obesity, or an increase in visceral adipose tissue (VAT), refers to fat tissue surrounding the organs, in contrast to subcutaneous adipose tissue. The accumulation of VAT plays a key role in the development of metabolic syndrome and is associated with an increased risk of cardiovascular diseases, including heart failure, coronary artery disease, pulmonary conditions, neurological disorders such as stroke, and cancer. Moreover, VAT is an independent predictor of mortality in men [18,19,20,21]. Patients with visceral fat accumulation have been shown to exhibit greater thickness of EAT compared to those with predominantly peripheral fat distribution [22,23].

EAT, as a manifestation of visceral fat, has itself been associated with an increased risk of atherosclerotic cardiovascular diseases, including coronary artery disease [24,25,26]. Several studies have identified EAT as a significant contributor to the development of atrial myopathy, atrial fibrillation, thromboembolic stroke, biventricular hypertrophy, and nervous system impairment, all of which are implicated in heart failure [23,27,28,29].

The characteristics of EAT have also been linked to major adverse cardiovascular events and elevated serum levels of inflammatory plaque markers. In fact, EAT has been shown to exert both paracrine and endocrine effects on other cardiac structures [30]. Notably, low-attenuation fat on CT—indicative of inflamed white adipose tissue—is more likely to influence the atherosclerotic process [28].

### 1.3. Radiomics and Texture Analysis

Radiomics using texture analysis (TA) is a promising approach that enables rapid extraction of specific features from biomedical images [31,32]. Machine learning and artificial intelligence algorithms can then be applied to derive insights into the underlying pathophysiology, diagnosis, and outcomes of many diseases [33,34,35,36].

TA involves identifying image features that are not detectable by the human eye and improving the diagnostic accuracy of radiological exams by using standardized mathematical formulas to calculate the relationships between neighboring voxels in terms of signal intensity. Radiomics has already shown promising results in the field of cardiac MRI, including distinguishing myocardial infarction from myocarditis, differentiating hypertrophic myocardium from normal myocardium and hypertrophic cardiomyopathy from hypertensive heart disease, predicting long-term outcomes in patients with Takotsubo syndrome, and detecting subtle cardiac changes related to ischemic processes [33,37,38,39]. There is growing interest, across multiple fields, in artificial intelligence systems that can predict structural abnormalities or the outcomes of pathological processes by analyzing intrinsic image features that are not detectable through conventional visual assessment.

### 1.4. Aim of the Study

The aim of the study is to investigate potential associations between EAT radiomics features and coronary artery non-calcified plaques with severe stenosis in patients with low to intermediate cardiovascular risk undergoing CCTA.

## 2. Materials and Methods

A retrospective study was carried out at University Hospital of Rome “Tor Vergata”, with approval from the ethics committee (R.S.71.21, protocol no. 5777).

### 2.1. Patients

The study was conducted on 128 patients with low to intermediate cardiovascular risk, according to the ESC 2021 Guidelines and the Heart Score, who had undergone CCTA with and without contrast agents for known or suspected coronary artery disease between September 2021 and May 2023. The patients provided written informed consent for the use of their demographic, clinical, and imaging data in anonymized form for research purposes. The inclusion and exclusion criteria for the study population are summarized in Table 1.

### 2.2. Coronary CT

CCTAs were performed with a 256-row wide-detector CT system (Revolution CT, GE HealthCare, Chicago, IL, USA) with 160 mm *z*-axis coverage and a gantry rotation time of 0.28 s, using retrospective gating in axial acquisition mode with ECG synchronization and high pitch, and images were reconstructed using ASiR. After baseline CT acquisition, a non-ionic low-osmolar contrast agent (Iomeprol 400 mgI/mL, Bracco Imaging S.p.A., Milan, Italy) was injected into an antecubital vein through a 20-gauge catheter, using a dual-head injector. All images were transferred to an external workstation (ADW version 4.7; GE HealthCare, Chicago, IL, USA) for post-processing analysis.

### 2.3. CT Image Processing

The CCT images were first screened using the Picture Archiving and Communication System provided by University Hospital of Rome “Tor Vergata”. The images were then classified based on the presence or absence of atherosclerotic plaques in the three main coronary arteries (left anterior descending artery, LAD; circumflex artery, Cx; right coronary artery, RCA). The classification considered the degree of stenosis and composition, with particular attention to the presence of low-density non-calcified plaques. For each plaque, the degree of stenosis was assessed using the CAD-RADS classification [40,41].

**Figure 1:**
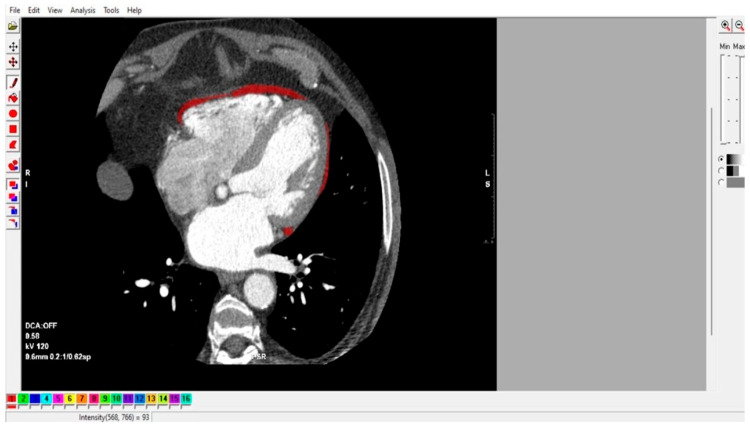
Placement of ROI on epicardial adipose tissue for radiomic feature extraction.

Using multiplanar reconstructions for each patient, a post-contrast injection four-chamber mesocardial view was obtained to enhance visualization of the epicardial adipose tissue. A single four-chamber view was selected to standardize the anatomical landmark and minimize *z*-axis motion artifacts common in volumetric EAT assessment. Each image was then segmented by a single expert operator with 6 years of experience in cardiovascular imaging using the open-source software MaZda version 4.6 (Institute of Electronics, University of Lodz, Lodz, Poland; accessed on 2 June 2025) [42], placing a region of interest (ROI) on the portions of EAT visible in the periventricular and pericoronary regions, as shown in Figure 1.

Grayscale was normalized by scaling the histogram data (mean ± 3 standard deviations) to reduce variations in contrast and intensity that could compromise the quantification of texture analysis features. A total of 337 features were extracted for each ROI. The extracted TA features included histogram analysis, gradient analysis, geometry, co-occurrence matrix features across five directions, run-length matrix features across four directions, autoregressive model features, and wavelet transformation. A detailed description of the radiomic features is freely available in the MaZda 4.6 online manual [42].

### 2.4. Processing of TA Data

Texture analysis data were normalized using an auto-scaling process (centered on the mean and divided by the standard deviation of each variable) and then processed using the Weka data mining platform (v3.8.5) [43], which, through preprocessing of the extracted radiomic features, helped reduce potential overfitting.

Features were selected to prioritize the highest correlation with the target variable and the lowest correlation among themselves. For the preliminary analysis, CfsSubsetEval was then used to identify a subset of features that were highly correlated with the presence of severe non-calcified plaque, while maintaining low inter-feature correlation.

Subsequently, the auto-scaled dataset was split 50:50 into a training set and a testing and validation set to ensure a robust size for the testing set. The former was used to train the classification models, and the latter to test and evaluate their performance. The classes were evenly balanced in each dataset.

Eleven classification models were formed: random forest (RF), neural network (Nnet), Naïve Bayes (NB), Extreme Gradient Boosting (XGB), Support Vector Machines (SVM), Support Vector Machines with linear kernel (svmLinear), k-nearest neighborhoods (knn), partial least squares (pls), decision tree (CART), logistic regression model (glm), and linear discriminant analysis (lda). An adaptive resampling cross-validation system (minimum number of resamples = 99, confidence level = 0.05) was used to prevent overfitting and ensure robust hyperparameter selection.

### 2.5. Ensemble Machine Learning

Models with a test set accuracy greater than 70% were selected and combined using an Ensemble Machine Learning (EML) model, which was based on averaging the obtained predictions.

To evaluate the EML’s ability to correctly classify the images, ROC curves were computed, along with sensitivity, specificity, positive and negative predictive values, and overall accuracy. The score that maximized Youden’s index (sensitivity + specificity − 1) was identified as the optimal threshold for distinguishing between the two classes.

To summarize the results, a confusion matrix was used to assess the overall diagnostic performance of the proposed scoring method.

### 2.6. Statistical Analysis

Statistical analysis was performed using R version 3.4.4 (R Core Team 2018. R: A language and environment for statistical computing. R Foundation for Statistical Computing, Vienna, Austria. URL https://www.R-project.org/ accessed on 2 June 2025) [44]. The packages used were “caret,” “stat,” “pROC,” and “OptimalCutpoints.” Categorical variables were displayed as absolute numbers and percentages, while continuous variables were shown as mean ± standard deviation. Continuous variables were compared using the Wilcoxon test. The Area Under the Curve (AUC) and ROC curves were used to compare the diagnostic performance of the various ML/AI models applied to each analyzed image. The AUCs obtained were compared using the DeLong test. The optimal threshold value was determined using Youden’s index. Additionally, thresholds were established to achieve a Negative Predictive Value (NPV) of 90% and a Positive Predictive Value (PPV) of 99%. The results showed that some values had a *p*-value lower than 0.05, indicating statistical significance.

## 3. Results

### 3.1. Study Population

**Scheme 1:**
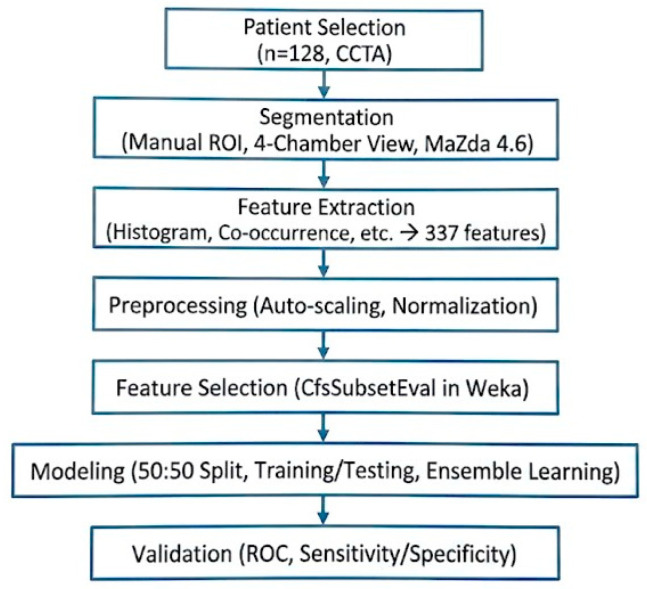
Study Workflow illustrating the radiomic pipeline from image segmentation to machine learning analysis.

The study population comprised 128 patients with low to intermediate cardiovascular risk who had undergone CCTA between September 2021 and May 2023. Their characteristics are summarized in Table 2. The workflow of data analysis is illustrated in Scheme 1.

### 3.2. Image Analysis

A total of 128 images obtained from cardiac CT scans were analyzed. The observed plaque characteristics and CAD-RADS scores are summarized in Table 3.

For each CCT exam, a single four-chamber image was segmented and analyzed for the assessment of epicardial adipose tissue using freehand ROI placement in MaZda 4.6. The selected variables are summarized in Table 4, which compares the mean values of each feature between patients with non-calcified plaques and CAD-RADS ≥ 4 and those without.

### 3.3. ROC Curve Analysis

The RF model showed a sensitivity of 0.66 and a specificity of 0.00, with a negative predictive value (NPV) of 0.00 and a positive predictive value (PPV) of 0.78, and an accuracy of 0.76 (95% CI: 0.383–0.713).

The NB model showed a sensitivity of 0.74 and a specificity of 0.43, with an NPV of 0.85 and a PPV of 0.30, and an accuracy of 0.68 (95% CI: 0.5135–0.825).

The SVM model showed a sensitivity of 0.66 and a specificity of 0.17, with an NPV of 0.20 and a PPV of 0.63, and an accuracy of 0.50 (95% CI: 0.3338–0.6662).

The linear SVM (SVM_L) model showed a sensitivity of 0.64 and a specificity of 0.10, with an NPV of 0.01 and a PPV of 0.67, and an accuracy of 0.50 (95% CI: 0.3338–0.6662).

The CART model showed a sensitivity of 0.70 and a specificity of 0.00, with an NPV of 0.00 and a PPV of 0.96, and an accuracy of 0.68 (95% CI: 0.5135–0.825).

The KNN model showed a sensitivity of 0.73 and a specificity of 0.32, with an NPV of 0.55 and a PPV of 0.55, and an accuracy of 0.53 (95% CI: 0.3582–0.6902).

The GLM model showed a sensitivity of 0.71 and a specificity of 0.00, with an NPV of 0.00 and a PPV of 0.00, and an accuracy of 0.71 (95% CI: 0.541–0.8458).

The PLS model showed a sensitivity of 0.70 and a specificity of 0.00, with an NPV of 0.96 and a PPV of 0.00, and an accuracy of 0.68 (95% CI: 0.5135–0.825).

The LDA model showed a sensitivity of 0.71 and a specificity of 0.00, with an NPV of 0.00 and a PPV of 0.00, and an accuracy of 0.71 (95% CI: 0.541–0.8458).

The NNET model showed a sensitivity of 0.65 and a specificity of 0.00, with an NPV of 0.00 and a PPV of 0.00, and an accuracy of 0.55 (95% CI: 0.383–0.7138).

The XGB model showed a sensitivity of 0.66 and a specificity of 0.00, with an NPV of 0.00 and a PPV of 0.78, and an accuracy of 0.55 (95% CI: 0.383–0.7138).

**Figure 2:**
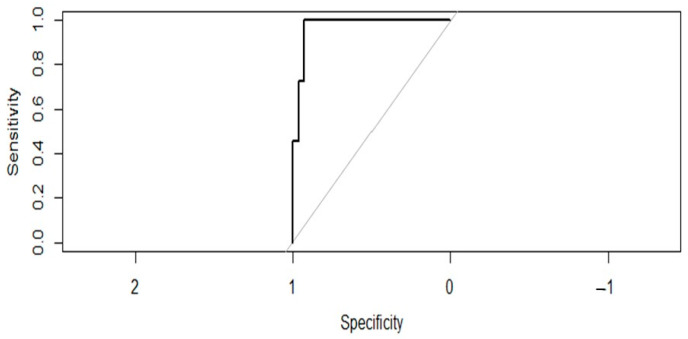
ROC curve of the EML model.

The EML model combined with coronary plaque data showed a sensitivity of 1.00 and a specificity of 0.93, with an NPV of 1.00 and a PPV of 0.85, and an accuracy of 0.95 (95% CI: 0.9221–1). The ROC curve is shown in Figure 2.

## 4. Discussion

### 4.1. Epicardial Adipose Tissue

An increasing body of research shows that epicardial adipose tissue not only serves as a protective mechanical cushion for the myocardium but also exerts biohumoral effects on both adjacent and non-adjacent cardiac structures. Goeller et al. and Abazid et al. have reported a correlation between EAD, coronary calcification (coronary calcium score), and the subclinical prevalence of CAD. These are just a few of the many studies exploring how EAT contributes to inflammation and plaque vulnerability, thereby elevating cardiovascular risk [16,24,27].

Low EAD is associated with BMI and higher CCS, suggesting the presence of chronic inflammation driven by white EAT that may elevate cardiovascular risk. Subjects with a CCS greater than 100 have a significantly lower EAD compared to those with a CCS of 0. Franssens et al. also described a link between low EAD and more extensive coronary calcification [28].

Research has shown that excess lipid accumulation in diet-induced obesity and insulin-resistant states is linked to adipose tissue characterized by lower CT attenuation with adipocyte hypertrophy and hyperplasia [28,45]. This adipose tissue secretes numerous molecules, such as plasminogen activator inhibitor-1 and monocyte chemoattractant protein-1, which are associated with low-grade systemic inflammation and heightened risk of vascular inflammation and atherosclerosis progression. Moreover, low-density EAT is associated with reduced levels of adiponectin, a protective factor against inflammation and atherogenesis, and therefore increases the risk of acute coronary syndrome and atherosclerosis progression [20].

Many inflammatory patterns linked to heart failure are caused by conditions such as obesity, hypertension, autoimmunity, and aging. Inflammatory biomolecules not only cause coronary microvascular endothelial inflammation, oxidative stress, reduced nitric oxide availability, and cardiomyocyte loss, but also directly affect cardiac immune cells, leading to local chronic inflammation [28]. The relationship between coronary atheromatosis and chronic inflammation suggests the potential to target these mediators therapeutically.

### 4.2. Radiomics

To our knowledge, this is the first study to use radiomic analysis of epicardial adipose tissue not only around the coronary arteries but also in the periventricular region to predict severe non-calcified coronary artery disease using the corresponding CAD-RADS score. There is growing interest in artificial intelligence systems that can predict the presence of structural abnormalities or the outcome of a pathological process by analyzing intrinsic imaging features that are not detectable through simple visual inspection [46,47].

Notably, machine learning and artificial intelligence tools are increasingly being used in diagnostic imaging. These technologies can analyze large volumes of data, which may consist of the pixels that make up the image or of intrinsic characteristics (radiomic features) extracted from the image itself. Radiomic feature analysis usually relies on expert operators, who play an active role in the data extraction, which can be a time-consuming process [48,49].

Studying radiomic features, backed by appropriate statistical analysis, also makes it possible to identify and potentially validate novel biomarkers for the diseases under investigation, which can be used for both diagnosis and prognosis. In this regard, a few emerging studies have begun to explore EAT radiomics and their prognostic implications [50,51,52,53]. Our work found a statistically significant correlation between specific radiomic features of epicardial adipose tissue and the presence of severe non-calcified coronary artery disease with CAD-RADS ≥ 4 and proposed the same kind of clinical applications.

### 4.3. Clinical Applications

If the results of this study are confirmed by further prospective research on larger populations, epicardial adipose tissue could be considered a new imaging biomarker for cardiovascular risk. Assessing the characteristics of EAT in patients undergoing chest CT, even without contrast agents, would allow for risk stratification and help determine which patients should be prioritized for further diagnostic investigations due to their higher risk of having management-relevant coronary artery disease.

Additionally, there are cases where a cardiac CT exam reveals moderate coronary pathology, and the radiologist may face the dilemma of whether to refer the patient for invasive follow-up through coronary angiography. Evaluation of EAT could aid in clinical decision-making for patients in these potential borderline cases.

## 5. Limitations of the Study

The main limitations of this study are its retrospective design, limited patient population, and the single-operator manual segmentation of epicardial adipose tissue on post-contrast images, although this approach did allow for the secure exclusion of coronary arteries and their branches. The retrospective design of the study prevented the collection of specific physiological parameters. As a result, a formal clinical risk stratification (e.g., via risk scores) could not be performed. Manual segmentation of EAT may introduce operator-dependent errors, as opposed to automated or semi-automated methods. The reliance on single-operator manual segmentation without inter-observer variability assessment (ICC) limits the generalizability of the results. Future research should employ volumetric segmentation and automated tools to enhance radiomic stability. Furthermore, while we categorized disease severity according to the CAD-RADS system, our analysis did not include specific stenosis percentages or a differentiation between CAD-RADS subgroups, which may carry distinct prognostic implications; future studies with larger cohorts should explore whether radiomic features can further stratify risk within these specific obstructive categories. Given that a substantial proportion of cardiovascular events arise from plaques causing less than 50% stenosis [3], future research involving larger cohorts across all CAD-RADS categories is needed to evaluate the broader predictive value of EAT radiomics. Additionally, while feature selection was performed to reduce dimensionality, the high number of radiomic features relative to the sample size poses a risk of overfitting. Although internal validation was performed, future studies utilizing nested cross-validation and larger datasets are required to robustly validate the stability of these radiomic signatures.

## 6. Conclusions

The EML model demonstrated excellent diagnostic accuracy in predicting the presence of non-calcified atheromatous plaques in the coronary circulation that result in severe stenosis, as determined from EAT radiomics in internal validation. These preliminary findings suggest that EAT radiomic features show a potential association with severe non-calcified coronary stenosis. This study is hypothesis-generating, and further prospective research with outcome-based validation is required to determine the clinical utility of this approach. and to determine if EAT characteristics may serve as a novel imaging biomarker for patients with low to intermediate cardiovascular risk.

## Figures and Tables

**Table 1:** Study population inclusion and exclusion criteria.

Inclusion Criteria	Exclusion Criteria
Low to moderate cardiovascular risk	History of coronary stent implantation or coronary artery bypass surgery
No history of acute coronary syndrome	Presence of valve prostheses
No history of heart surgery	High cardiovascular risk
No history of valvular disease	History of acute coronary syndrome Presence of valvular disease

**Table 2:** Clinical characteristics of the study population.

**Clinical Characteristics**	**Mean ± DS/N (%)**
Age	52.3 ± 13.3
Gender [Female]	52 (41%)
Hypertension	75 (59%)
Smoking	56 (44%)
Diabetes	37 (29%)
Dyslipidemia	64 (50%)
**CAD-RADS**	**N (%)**
0	13 (10.2%)
1	13 (10.2%)
2	27 (21.1%)
3	33 (25.8%)
4 A/B	36 (28.1%)
5	6 (4.7%)

**Table 3:** Plaque characteristics of the study population.

Plaque Characteristics	N (%)
CAD-RADS ≥ 4 Of which: non-calcified	42 (32.81%) 24 (18.74%)
Plaque in left anterior descending artery Of which: non-calcified	110 (85.9%) 16 (12.49%)
Plaque in right coronary artery Of which: non-calcified	87 (68.0%) 9 (7.03%)
Plaque in circumflex artery Of which: non-calcified	86 (67.2%) 6 (4.69%)

**Table 4:** Variables (expressed as mean values) selected based on their inter-correlation and correlation with the target variable.

	CAD-RADS ≥ 4, Non-Calcified	Other Patients	*p*-Value
GeoLminE	57,233,672	45,722,331	0.0025
GeoRm	17,398,473	16,123,791	0.82
S.0.2.Entropy	28,376,187	26,637,187	0.0004
S.3..3.SumVarnc	27,117,218	28,181,723	0.029
S.3..3.Entropy	24,817,123	21,239,818	0.0003
X_Area_S.4.4.	5387.273	5123.9854	0.14
Vertl_LngREmph	13,189,847	14,983,428	0.27
WavEnHL_s.1	58,778,521	56,187,231	0.0006
WavEnHH_s.1	44,328,818	47,827,341	0.015

## Data Availability

Data are available under reasonable request to the corresponding author.

## Abbreviations

The following abbreviations are used in this manuscript:

EAT	Epicardial Adipose Tissue
TA	Texture Analysis
CAD	Coronary Artery Disease
CCTA	Coronary Computed Tomography Angiography
CCS	Coronary Calcium Score
AUC	Area Under the Curve
ROC	Receiving Operator Curve
EML	Ensemble Machine Learning Model
RF	Random Forest
NNET	Neural Network
NB	Naïve Bayes
XGB	Extreme Gradient Boosting
SVM	Support Vector Machines
SVMl	Support Vector Machines with linear kernel
CART	Decision Tree
GLM	Logistic Regression Model
KNN	K-Nearest Neighborhoods
LDA	Linear Discriminant Analysis
